# Effect of speed and gradient on plantar force when running on an AlterG® treadmill

**DOI:** 10.1186/s13102-021-00258-4

**Published:** 2021-03-30

**Authors:** Athol Thomson, Rodney Whiteley, Clint Hansen, Julius Welzel, Sebastien Racinais, Mathew G. Wilson

**Affiliations:** 1 Aspetar Orthopaedic & Sports Medicine Hospital, PO Box 29222, Doha, Qatar; 2 Discipline of Podiatry, School of Allied health, La Trobe University, Melbourne, Victoria 3086 Australia; 3 Kiel University Department of Neurology, UKSH campus, 24105 Kiel, Germany; 4 Institute of Sport, Exercise and Health, University College London, London, UK

**Keywords:** Plantar force, In-shoe force, Rehabilitation, Running, Biomechanics

## Abstract

**Background:**

Anti-gravity treadmills are used to decrease musculoskeletal loading during treadmill running often in return to play rehabilitation programs. The effect different gradients (uphill/downhill running) have on kinetics and spatiotemporal parameters when using an AlterG® treadmill is unclear with previous research focused on level running only.

**Methods:**

Ten well-trained healthy male running athletes ran on the AlterG® treadmill at varying combinations of bodyweight support (60, 80, and 100% BW), speed (12 km/hr., 15 km/hr., 18 km/hr., 21 km/hr., and 24 km/hr), and gradients (− 15% decline, − 10, − 5, 0, + 5, + 10 + 15% incline), representing a total of 78 conditions performed in random order. Maximum plantar force and contact time were recorded using a wireless in-shoe force sensor insole system.

**Results:**

Regression analysis showed a linear relationship for maximum plantar force with bodyweight support and running speeds for level running (*p* < 0.0001, adj. R^2^ = 0.604). The linear relationship, however, does not hold for negative gradients at speeds 12 & 15 km/h, with a relative ‘dip’ in maximum plantar force across all assisted bodyweight settings.

**Conclusions:**

Maximum plantar force peaks are larger with faster running and smaller with more AlterG® assisted bodyweight support (athlete unweighing). Gradient made little difference except for a downhill grade of − 5% decreasing force peaks as compared to level or uphill running.

**Supplementary Information:**

The online version contains supplementary material available at 10.1186/s13102-021-00258-4.

## Background

Graduated return to weight-bearing activity forms a mainstay in the management of many lower extremity injuries [1–3]. Progression of the optimal level of load is fundamental to maximising physiological adaptation while preventing excessive overload during rehabilitation [3–5]. Increasingly, reduced gravity treadmills are being utilised for rehabilitation of lower extremity injuries to manipulate the magnitude of load on the musculoskeletal system while walking or running [6–8].

AlterG® treadmill use is widespread across elite sports clubs and physical rehabilitation clinics worldwide (AlterG®, California USA). Athletes wear neoprene shorts zipped into a sealed chamber surrounding the treadmill while positive air pressure is pumped into the chamber to reduce the athlete’s bodyweight (BW). The amount of reduction in athlete BW used can range from no assistance (100% of athletes’ BW) down to 20% (i.e. extra positive air pressure pumped into chamber to lift 80% of the athlete’s BW off the treadmill deck).

Manipulation of running speed and/or AlterG® assisted BW support affects the magnitude of vertical ground reaction force or maximum plantar force (Fmax) experienced by athletes running on the treadmill. A linear relationship exists for slow to moderate running speeds (10-17 km/hr) whereby increasing running speed leads to increased Fmax and loading rate. Conversely, Fmax and loading rate decrease linearly as AlterG® assisted BW support is boosted to unweigh the athlete [9–11]. However, previous studies have examined level running (no inclines/declines) on a treadmill with a focus on steady-state running speeds (10-17 km/hr). The effect different treadmill gradients (inclines/declines) may have on plantar loading parameters while running in an AlterG® treadmill is yet to be studied.

Wireless force sensing insoles are novel low-cost alternative to traditional embedded force plates for collection of clinical and research data. Loadsol® (Novel, Munich, Germany) insoles measure vertical ground reaction force experienced at the plantar surface of the foot (and contact times) during stance phase of gait, with excellent validity and reproducibility for walking, running [12, 13], jumping, and hopping [14, 15]. One advantage of wireless force sensing insoles is the ability to collect data on multiple steps when running in varied locations such as the field of play or in reduced gravity treadmills.

Grade specific biomechanical adaptations occur for uphill and downhill running. These include changes to foot-strike pattern, ground reaction forces, joint kinematics, and tibial accelerations [16]. No studies have investigated these changes on an anti-gravity treadmill.

Our aim is to quantify plantar loading parameters across a range of inclines, declines, running speeds, and AlterG® bodyweight assistance (unweighting) settings while on a reduced gravity treadmill and compare them to level running using a wireless insole system.

## Methods

### Participants

Ten healthy (free from lower limb injury for past 6 months) well trained male athletes volunteered to take part in this study (age 28 ± 5 yrs., weight 73 ± 8 kg, height 180 ± 6 cm). Informed consent was obtained for each participant, following approval of the local ethics committee (Anti-Doping Lab Qatar Approval number #E2018000272). The sample size was selected based on pilot data collected on two subjects, and calculated in G*Power (Universität Düsseldorf, Germany). To create a conservative estimate of sample size, a low correlation of 0.1 was assumed between conditions, and a one-tailed 95% confidence interval was selected. This resulted in a calculated sample size of 8. Hence, an estimation of 10 subjects in this study was recommended for potential or unexpected data or subject dropout.

Participants regularly ran at speeds greater than 24 km/hr. and in the 3-months leading into the study, ran on average 31 ± 15 km per week (self-reported). All participants were familiar with treadmill running usually completing a minimum of one treadmill session per week in the 3 months prior.

### Equipment

Plantar loading parameters were measured using an in-shoe load monitoring device (Loadsol®, Novel, Munich, Germany). Each Loadsol® insole consists of two capacitive force sensors that transmit data over Bluetooth to a smartphone or tablet. Force sensor insoles were placed inside the participants own preferred running shoes in the appropriate size. No participants used orthotic supports. Insole calibration followed manufacturers guidelines (Novel, Munich, Germany) with calibration accepted if a bodyweight ±5% of the athletes’ bodyweight was achieved at single leg stance with the insoles fully loaded. Insole resolution was set at 5/10Newtons for a range of 0-2550 N and a sample rate of 200 Hz using the Loadsol® App (version 1.5.10) on an Apple Ipad Pro 9.7 in. (Apple, Cupertino, USA). Here, we use the most recent generation of Loadsol® insoles with a sample rate of 200 Hz which demonstrated improved validity (ICC 0.76–0.98) over the previous generation of insoles (100 Hz) when compared with a force plate sampling at 1920 Hz [15]. Loadsol® insoles sampling at 200 Hz underestimate vertical force measurements in a reliable way when compared to force plates (95% limits of agreement 0.02 to 0.69BW) [12, 15].

### Warm-up protocol

Participants were fitted with the correct sized AlterG® shorts. A calibration protocol according to the manufacturer instructions was followed whereby the athlete stands with arms folded across their chest while the bodyweight of the athlete is measured on the treadmill deck (G-trainer pro 2.0, AlterG®, California USA). All participants used the same warm-up protocol: Walk for 5 min at 5 km/hr. at 100% bodyweight (No AlterG® assisted BW support), Run for 3 min at 10 km/hr. at 100% of BW. Followed by 2 × 10 s efforts at 21 km/hr. and 2 × 10 s efforts at 24 km/hr. at 100% BW (with 30 s static recovery in between efforts), in order to familiarize themselves with getting on and off the treadmill at high speed.

### Testing protocol

Following warm-up participants ran at varying combinations of AlterG® indicated BW support (60, 80, and 100% BW), speed (12 km/hr., 15 km/hr., 18 km/hr., 21 km/hr., and 24 km/hr), and gradients (− 15% decline, − 10, − 5, 0, + 5, + 10 + 15% incline) for approximately 60 s per trial. Sum of total running trials was 78 with all possible combinations. Each of these combinations of speed, gradient and BW was block randomised a priori using online software (www.randomizer.org). A recovery period was set at a minimum of 45 s between each trial. All data was collected at a single visit for each participant.

For the downhill running trials, participants faced ‘backwards’ in the AlterG® treadmill and the belt was run in reverse. Hence, the inline function with belt in reverse direction can be used as a decline when facing away from the usual running direction. Top speed for the treadmill (G-trainer pro 2.0, AlterG®, California USA) in reverse was 15 km/hr., therefore, all decline conditions were conducted at two running speeds of 12 &15 km/hr.

For each trial condition participants were instructed to run until they felt comfortable, and then indicate the point where their gait felt “normal”. Loadsol® insole data was then collected at 200 Hz for a minimum of 6 stance phase foot contacts of both feet. Threshold of 30 N was set to identify when stance phase commenced to decrease any signal noise associated with treadmill running.

### Statistical & data analysis

All data were processed using custom scripts(https://github.com/JuliusWelzel/AlterG-loadsol) for MATLAB (Version 9.6; MathWorks, Natick, MA, USA). Maximum plantar force (Fmax) for each foot were extracted respectively from the time of stance and averaged for subsequent analysis over a minimum of six footfalls for each foot (twelve total). Outliers in the data excluded elements more than 1.5 interquartile ranges above the upper quartile or below the lower quartile [17]. Single outliers in footfalls were removed leaving a minimum of five footfalls for analysis. Maximum force was normalised to participants bodyweights to aid comparison across the group. Multiple linear regression was used to reveal the relationship between running speed, percentage body weight and normalized maximal plantar force as outcome variable. To understand the effect of different gradients during running on the loading forces, another multiple linear regression was conducted with running speed, AlterG® assisted BW support, and gradient as regressors. Post-hoc analysis used repeated measures ANOVA with Bonferroni correction and subsequent pairwise comparisons and effect size calculations. Level of significance was set a priori at *p* = 0.05. Effect sizes (cohen’s d) were reported as small, medium, large, and very large when they reached 0.2, 0.5, 0.8, 1.2 respectively [18, 19]. The maximum plantar force data collected by the Loadsol® insoles are reported in units of BW (times bodyweight). The indicated AlterG® bodyweight support on the treadmill is reported as percentage of bodyweight (%BW). Treadmill incline or decline is reported as a gradient (%). Contact time is reported in milliseconds (ms).

## Results

### Maximum plantar force

**Fig. 1:**
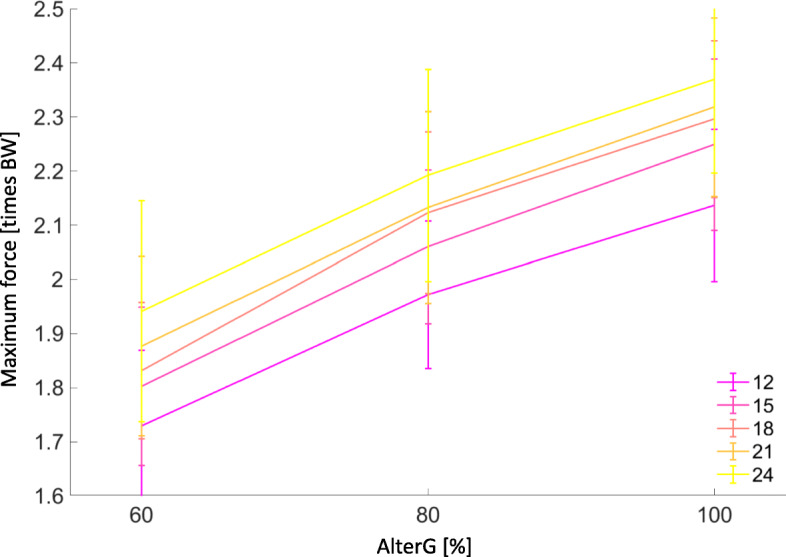
Maximum plantar force [times BW] mean ± Standard deviation (error bars) at different running speeds and AlterG® assisted bodyweight support [%] shows a linear relationship. (Adj. R^2^: 0.604, *p* < 0.001)

Regression analysis showed a linear relationship for Fmax by AlterG® assisted BW support and different (faster) running speeds for level running (*p* < 0.001, adj. R^2^ = 0.604) (Fig. 1).

$$ Regression\ model: Peak\ force= 1+ AlterG\ bodyweight+\mathit{\log}\ (speed) $$

Multiple regression analysis shows that the relationship between Fmax, AlterG® assisted BW support, running speeds, including multiple gradients of running, remains linear for positive gradients only (*p* < 0.001, adj R^2^ = 0.613) (Supplementary figure 1)

Fmax was highest at 24 km/hr., level gradient, at 100% AlterG BW measured at 2.46 ± 0.2 times BW and lowest at 12 km/hr., − 5% decline gradient, at 60% AlterG BW measured at 1.65 ± 0.2 times BW (*p* < 0.001, d = 4.05).

**Fig. 2:**
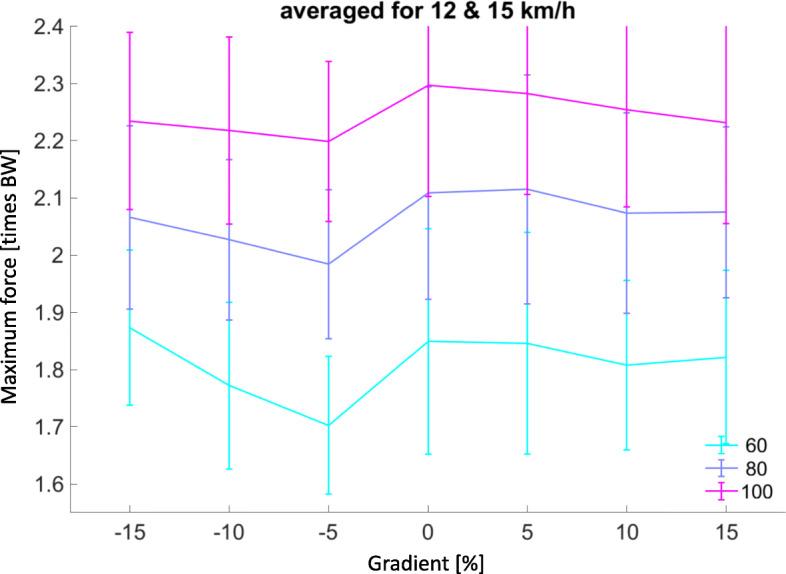
Maximum plantar force [times BW] mean ± standard deviations (error bars) varies with gradient for speeds 12 & 15 km/hr. averaged. 15 km/hr. was the top speed when running the treadmill belt in reverse to get the decline trials. Therefore, the common speeds use for both incline and decline conditions have been averaged for comparison. Note non-linear relationship for negative gradient with a relative dip for the − 5° running trials

There was a significant decrease in Fmax with bodyweight unloading (Fig. 2 & supplementary figure 2). For example, at the fastest speed of 24 km/hr., level gradient, at 100% AlterG BW measured at 2.46 ± 0.2 times BW compared to 24 km/hr., level gradient, at 60% AlterG BW measured at 1.99 ± 0.3 times BW (*p* < 0.001, d = 1.84). To visualise pairwise comparisons across all running trial conditions, an online platform has been created at (https://secret-wave-84791.herokuapp.com/). Post-hoc comparisons are also presented in supplementary table 1.

The linear relationship, however, does not hold for negative gradients at speeds 12 & 15 km/h (Fig. 2), with a relative ‘dip’ in Fmax across all AlterG® assisted BWs.

### Contact time

**Fig. 3:**
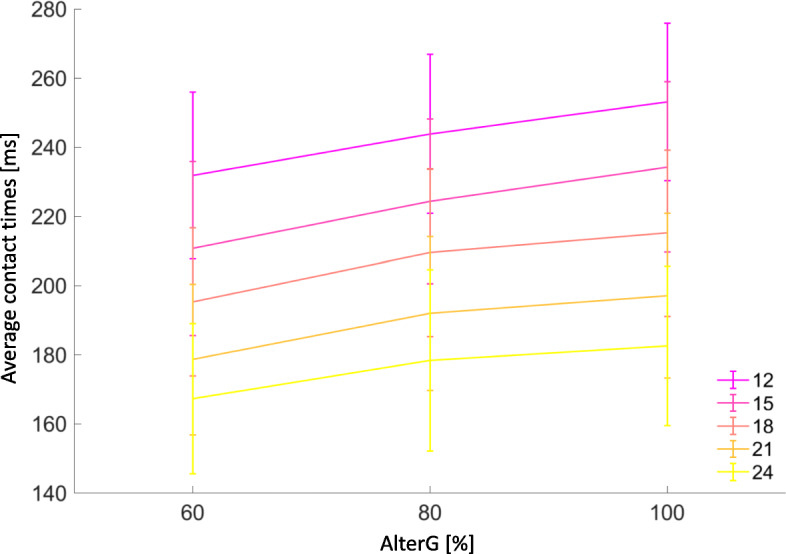
Average contact times [ms] ± SD (error bars) at different speeds and AlterG® assisted bodyweight support (adj. R^2^0.535, *p* < 0.001)

Regression analysis showed a linear relationship for contact times (*p* < 0.001, adj. R^2^ = 0.533) by AlterG® assisted BW support, and different running speeds (Fig. 3 and supplementary figure 2 & 3). Contact times decreased with faster running speed, and more AlterG® assisted BW support with level treadmill gradient.

$$ Regression\ model: Contact\ time= 1+ AlterG\ Bodyweight+\mathit{\log}(speed) $$

**Fig. 4:**
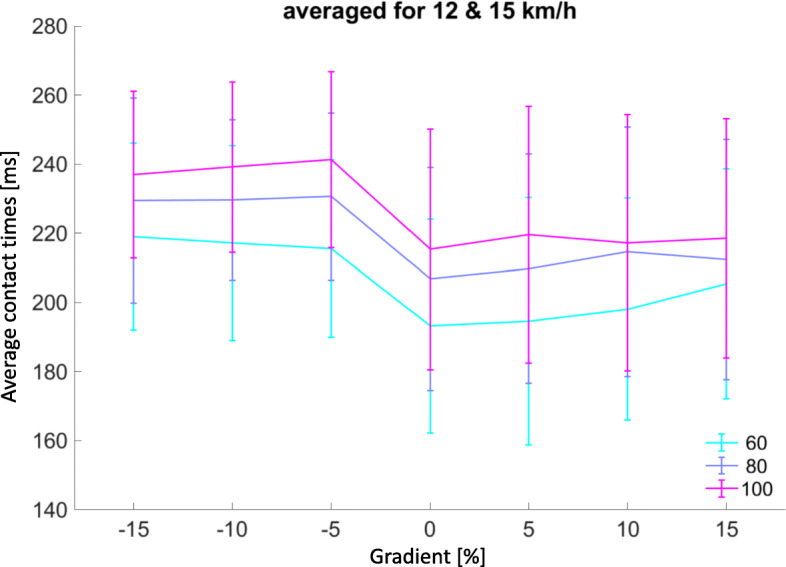
Average contact times [ms] mean ± SD (error bars) vary with gradient for speeds 12 & 15 km/hr. averaged. 15 km/hr. was the top speed when running the treadmill belt in reverse to get the decline trials. Therefore, the common speeds use for both incline and decline conditions have been averaged for comparison

Multiple linear regression including gradient showed a linear relationship for contact time (*p* < 0.001, adj. R^2^ = 0.542) (supplementary figure 4). However, again the linear relationship does not hold for negative gradients at speed of 12 & 15 km/h (Fig. 4). A relative increase in contact times was noted at the − 5% decline gradient across all AlterG® assisted BW support at these speeds.

## Discussion

Our study indicates running speed, treadmill decline, and AlterG® assisted BW support settings all have an effect on plantar force and contact times when running at speeds from 12 to 24 km/hr. Maximum plantar force peaks are larger with faster running and smaller with more AlterG® assisted BW support (athlete unweighing). Gradient made little difference except at the initial downhill grade of − 5% compared to level running where we observed a decrease in force peaks.

### Plantar force

Maximum plantar force (Fmax) is a measure of in-shoe ground reaction force experienced perpendicular to the plantar surface of the foot. We observed Fmax increased linearly with faster running speeds at all levels of weight support during level running (no gradient). These findings are similar to previous studies conducted at slower running speeds (< 17 km/hr) [10, 11] and the only previous study at faster speeds (up to 22 km/hr) [20] done on level treadmill setting. Ankle plantarflexors, including soleus and gastrocnemius, contribute most significantly to vertical support forces during slow and medium-paced level running up to 25 km/hr. [21]. This may explain the linear relationship between Fmax and speed here. Dorn and colleagues [21] report a shift in muscular recruitment strategies when running above 25 km/hr. to increased utilisation of hip musculature. During pilot testing participants found it difficult to run any faster than 24 km/hr. on the treadmill with positive gradients up to + 15°. Hence, we capped the speed at 24 km/hr.

Our study is the first (to our knowledge) to examine AlterG® treadmill running on different gradients with some novel findings. Downhill running showed a decrease in Fmax when running at 12 and 15 km/hr. on a − 5% decline gradient across all AlterG® assisted BW support settings (60,80, 100% BW). This finding is in contrast to previous research on regular treadmill running [22, 23]. Gottschall & Kram [22] found no significant variation in active (propulsive) vertical ground reaction force peaks for similar gradients (±5.2, 10.5, and 15.7%) in a cohort of healthy recreational male and female runners (*n* = 10). However, they found impact (braking) force peaks and initial loading rate to increase significantly with downhill running. Our study examined maximum force peaks (impact or active) and found active peaks to be higher across all the runners in this cohort when using the AlterG® treadmill. Our findings may indicate a unique set of conditions when downhill running on an AlterG® treadmill that are likely related to the supporting frame of the chamber athletes are zipped into, especially when positive air pressure is added to ‘lift’ the athlete off the treadmill deck. Location of force measurement (in-shoe vs force plate under treadmill) is probable another reason for this divergence.

Gottschall & Kram [22] also analysed horizontal (parallel to treadmill) force peaks and found a significant increase in horizontal braking forces of 27% for downhill running at − 5.2% and a 73% increase at − 15% downhill gradient compared to level running. In contrast, horizontal propulsive force peaks decreased by 22% for downhill − 5% gradient and 61% for downhill − 15% gradient. Hence, further research is required to examine horizontal force peaks during graded running on the AlterG® treadmill to elucidate any relationship with vertical force peaks examined here.

This new finding may have implications when looking to decrease maximum plantar force for rehabilitation. Rearfoot strike pattern is common with downhill running and less AlterG® assisted BW support (> 80% athlete BW setting) [10, 22]. We suggest that manipulation of gradient, running speed and using AlterG® assisted BW > 80% BW may be useful when attempting to decrease load at the forefoot (metatarsal fracture rehabilitation for example). Of course, this comes with the caveat that load will likely be shifted to other structures such as the ankle, knee, or hip.

Significant biomechanical and physiological alterations are evident with bodyweight support settings < 70% on the AlterG® with some authors suggesting staying above this threshold for to minimise changes in running mechanics [24–26]. Individual responses to our difference running trial conditions were somewhat variable. We suggest using supplementary figures 2, 3 and 4 to asses individual responses along with the mean and standard deviation ‘group’ data presented in the results section.

### Contact time

We saw a relative increase in contact times for downhill running at − 5% gradient across all AlterG® assisted BW support at these speeds (Fig. 4). This too is in contrast to previous research on regular treadmill running where no significant variation in contact times were seen for uphill or downhill running [22]. Again, these findings may indicate a unique set of conditions when downhill running on an AlterG® treadmill that are likely related to the supporting frame of the chamber athletes are zipped into, especially when positive air pressure is added to ‘lift’ the athlete off the treadmill deck. Location of force measurement (in-shoe vs force plate under treadmill) is probable another reason for this divergence.

Our previous research found walking and running speed vary contact times more than different AlterG® assisted BW support settings [11]. These running speeds concur with those findings.

### Limitations

There are a number of limitations to this study. Collection frequency of 200 Hz could result in the loss of true peak value for Fmax. In-shoe force measurement gives the vertical component of ground reaction force only and therefore does not capture medial-lateral or horizontal “shear” force that may be important components when considering lower extremity injury [22]. Increases in GRF metrics are not a direct indicator of increases of loading on internal structures such as the tibia bone [26, 27]. Therefore increases in GRF metrics in isolation may not mean increased running-related overuse injury risk. Ueberschär et al. [26] report no reduction in peak tibial impact/push-off acceleration magnitudes when running in hypogravity even though vertical ground reaction force is diminished.

Vertical ground reaction force or maximum plantar force examined here give a ‘global’ measurement of the impact and/or active peak force between the treadmill deck and the foot. Hence, no inferences are possible concerning how muscular recruitment strategies, joint torques, or regional loading parameters may shift under the different trial conditions examined here. Caution should be exercised when comparing these Fmax from AlterG® treadmill running to over-ground running as it is known that loads increase with over-ground running and this may result in an under-estimation of Fmax at the given running speed [28].

Little is known about measurement of in-shoe (vertical or perpendicular) plantar force when running on steep inclines or declines. This should be considered when evaluating the results here.

Finally, this study was conducted in healthy adult male subjects wearing their preferred footwear, it is unknown if the findings would be replicated in injured subjects, women, or children where gait parameters will likely vary.

## Conclusions

Maximum plantar force peaks are larger with faster running and smaller with more AlterG® assisted BW support (athlete unweighing). Gradient made little difference except at the initial downhill grade of − 5% compared to level running where we observed a significant decrease in force peaks. Further research is required to examine individual joint kinetics or muscle activation strategies for graded running on an AlterG® treadmill.

## Supplementary Information

**Additional file 1: Table S1**. Post-hoc comparisons for both Fmax and contact time across the different speeds, BW support, and gradients.

**Additional file 2: Figure S1.** Multiple linear regression including gradient for Maximum plantar force [times BW] at different running speed [km/hr], gradients [%], and levels of AlterG® assisted bodyweight support [%]. (adj. R^2^: 0.613, *p* < 0.001).

**Additional file 3: Figure S2.** Individual data for maximum plantar force [times BW] and contact times at different AlterG® assisted bodyweight support.

**Additional file 4: Figure S3.** Individual data for maximum plantar force [times BW] and contact times at different running speeds.

**Additional file 5: Figure S4.** Multiple linear regression including gradient for average contact times. (adj. R^2^: 0.52, *p* < 0.001).

**Additional file 6.** Data Set.

## Data Availability

An online platform has been created at to compare conditions for all combinations or running speed, assisted bodyweight, and gradient (https://secret-wave-84791.herokuapp.com/). Custom written Matlab scripts are available at. The datasets used and/or analysed are available from the corresponding author on request.

## Abbreviations

BW: Bodyweight
Fmax: Maximum plantar force
Hz: Hertz
