# Supplementary material for: Effect of speed and gradient on plantar force when running on an AlterG® treadmill
Source: BMC Sports Sci Med Rehabil. 2021 Mar 30;13:34. doi: 10.1186/s13102-021-00258-4 (PMC8011121; doi:10.1186/s13102-021-00258-4)
Supplement: Supplementary file 1 — Additional file 1: Table S1. Post-hoc comparisons for both Fmax and contact time across the different speeds, BW support, and gradients. [file 13102_2021_258_MOESM1_ESM.docx]

**Post Hoc Tests - Fmax**

| **Post Hoc Comparisons - speed** | | | | | | | | | | | |
| --- | --- | --- | --- | --- | --- | --- | --- | --- | --- | --- | --- |
|  | |  | | **Mean Difference** | | **SE** | | **t** | | **p _bonf_** | |
| 12 |  | 15 |  | -0.094 |  | 0.020 |  | -4.610 |  | < .001 |  |
|  |  | 18 |  | -0.152 |  | 0.020 |  | -7.408 |  | < .001 |  |
|  |  | 21 |  | -0.178 |  | 0.021 |  | -8.660 |  | < .001 |  |
|  |  | 24 |  | -0.239 |  | 0.021 |  | -11.647 |  | < .001 |  |
| 15 |  | 18 |  | -0.057 |  | 0.020 |  | -2.803 |  | 0.052 |  |
|  |  | 21 |  | -0.083 |  | 0.020 |  | -4.068 |  | < .001 |  |
|  |  | 24 |  | -0.145 |  | 0.020 |  | -7.061 |  | < .001 |  |
| 18 |  | 21 |  | -0.026 |  | 0.020 |  | -1.271 |  | 1.000 |  |
|  |  | 24 |  | -0.087 |  | 0.020 |  | -4.264 |  | < .001 |  |
| 21 |  | 24 |  | -0.061 |  | 0.021 |  | -2.987 |  | 0.029 |  |
|  | | | | | | | | | | | |
| *Note.*  P-value adjusted for comparing a family of 5 | | | | | | | | | | | |
| *Note.*  Results are averaged over the levels of: gradient, bw | | | | | | | | | | | |

| **Post Hoc Comparisons - BW** | | | | | | | | | | | |
| --- | --- | --- | --- | --- | --- | --- | --- | --- | --- | --- | --- |
|  | |  | | **Mean Difference** | | **SE** | | **t** | | **p _bonf_** | |
| 60 |  | 80 |  | -0.262 |  | 0.016 |  | -16.486 |  | < .001 |  |
|  |  | 100 |  | -0.438 |  | 0.016 |  | -27.589 |  | < .001 |  |
| 80 |  | 100 |  | -0.176 |  | 0.016 |  | -11.054 |  | < .001 |  |
|  | | | | | | | | | | | |
| *Note.*  P-value adjusted for comparing a family of 3 | | | | | | | | | | | |
| *Note.*  Results are averaged over the levels of: speed, gradient | | | | | | | | | | | |

| **Post Hoc Comparisons - gradient** | | | | | | | | | | | |
| --- | --- | --- | --- | --- | --- | --- | --- | --- | --- | --- | --- |
|  | |  | | **Mean Difference** | | **SE** | | **t** | | **p _bonf_** | |
| -15 |  | -10 |  | 0.053 |  | 0.026 |  | 2.017 |  | 0.932 |  |
|  |  | -5 |  | 0.097 |  | 0.026 |  | 3.738 |  | 0.004 |  |
|  |  | 0 |  | 0.077 |  | 0.026 |  | 2.980 |  | 0.064 |  |
|  |  | 5 |  | 0.078 |  | 0.026 |  | 2.986 |  | 0.063 |  |
|  |  | 10 |  | 0.085 |  | 0.026 |  | 3.255 |  | 0.026 |  |
|  |  | 15 |  | 0.080 |  | 0.026 |  | 3.068 |  | 0.048 |  |
| -10 |  | -5 |  | 0.045 |  | 0.026 |  | 1.713 |  | 1.000 |  |
|  |  | 0 |  | 0.025 |  | 0.026 |  | 0.955 |  | 1.000 |  |
|  |  | 5 |  | 0.025 |  | 0.026 |  | 0.961 |  | 1.000 |  |
|  |  | 10 |  | 0.032 |  | 0.026 |  | 1.230 |  | 1.000 |  |
|  |  | 15 |  | 0.027 |  | 0.026 |  | 1.051 |  | 1.000 |  |
| -5 |  | 0 |  | -0.020 |  | 0.026 |  | -0.762 |  | 1.000 |  |
|  |  | 5 |  | -0.020 |  | 0.026 |  | -0.756 |  | 1.000 |  |
|  |  | 10 |  | -0.013 |  | 0.026 |  | -0.485 |  | 1.000 |  |
|  |  | 15 |  | -0.017 |  | 0.026 |  | -0.658 |  | 1.000 |  |
| 0 |  | 5 |  | 0.007 |  | 0.018 |  | 0.362 |  | 1.000 |  |
|  |  | 10 |  | 0.042 |  | 0.018 |  | 2.316 |  | 0.125 |  |
|  |  | 15 |  | 0.046 |  | 0.018 |  | 2.519 |  | 0.072 |  |
| 5 |  | 10 |  | 0.036 |  | 0.018 |  | 1.951 |  | 0.309 |  |
|  |  | 15 |  | 0.040 |  | 0.018 |  | 2.153 |  | 0.190 |  |
| 10 |  | 15 |  | 0.004 |  | 0.018 |  | 0.202 |  | 1.000 |  |
|  | | | | | | | | | | | |
| *Note.*  P-value adjusted for comparing a family of 7 | | | | | | | | | | | |
| *Note.*  Results are averaged over the levels of: speed, bw | | | | | | | | | | | |

**Post Hoc Tests – Contact Time**

| **Post Hoc Comparisons - gradient** | | | | | | | | | | | | | | | |
| --- | --- | --- | --- | --- | --- | --- | --- | --- | --- | --- | --- | --- | --- | --- | --- |
|  | | | | | | **95% CI for Mean Difference** | | | |  | | | | | |
|  | |  | | **Mean Difference** | | **Lower** | | **Upper** | | **SE** | | **t** | | **p _bonf_** | |
| -15 |  | -10 |  | -0.331 |  | -13.354 |  | 12.692 |  | 4.395 |  | -0.075 |  | 1.000 |  |
|  |  | -5 |  | -1.030 |  | -14.108 |  | 12.049 |  | 4.414 |  | -0.233 |  | 1.000 |  |
|  |  | 0 |  | -5.004 |  | -18.082 |  | 8.074 |  | 4.413 |  | -1.134 |  | 1.000 |  |
|  |  | 5 |  | -7.198 |  | -20.221 |  | 5.825 |  | 4.395 |  | -1.638 |  | 1.000 |  |
|  |  | 10 |  | -8.565 |  | -21.588 |  | 4.459 |  | 4.395 |  | -1.949 |  | 1.000 |  |
|  |  | 15 |  | -12.411 |  | -25.490 |  | 0.668 |  | 4.414 |  | -2.812 |  | 0.108 |  |
| -10 |  | -5 |  | -0.698 |  | -13.722 |  | 12.325 |  | 4.395 |  | -0.159 |  | 1.000 |  |
|  |  | 0 |  | -4.673 |  | -17.696 |  | 8.350 |  | 4.395 |  | -1.063 |  | 1.000 |  |
|  |  | 5 |  | -6.867 |  | -19.835 |  | 6.101 |  | 4.376 |  | -1.569 |  | 1.000 |  |
|  |  | 10 |  | -8.233 |  | -21.201 |  | 4.735 |  | 4.376 |  | -1.881 |  | 1.000 |  |
|  |  | 15 |  | -12.080 |  | -25.103 |  | 0.943 |  | 4.395 |  | -2.749 |  | 0.131 |  |
| -5 |  | 0 |  | -3.975 |  | -17.053 |  | 9.104 |  | 4.414 |  | -0.901 |  | 1.000 |  |
|  |  | 5 |  | -6.168 |  | -19.191 |  | 6.855 |  | 4.395 |  | -1.403 |  | 1.000 |  |
|  |  | 10 |  | -7.535 |  | -20.558 |  | 5.488 |  | 4.395 |  | -1.714 |  | 1.000 |  |
|  |  | 15 |  | -11.381 |  | -24.459 |  | 1.696 |  | 4.413 |  | -2.579 |  | 0.216 |  |
| 0 |  | 5 |  | -1.784 |  | -8.800 |  | 5.231 |  | 2.723 |  | -0.655 |  | 1.000 |  |
|  |  | 10 |  | -3.569 |  | -10.596 |  | 3.459 |  | 2.728 |  | -1.308 |  | 1.000 |  |
|  |  | 15 |  | -6.168 |  | -13.196 |  | 0.859 |  | 2.728 |  | -2.261 |  | 0.145 |  |
| 5 |  | 10 |  | -1.784 |  | -8.800 |  | 5.232 |  | 2.723 |  | -0.655 |  | 1.000 |  |
|  |  | 15 |  | -4.384 |  | -11.400 |  | 2.632 |  | 2.723 |  | -1.610 |  | 0.648 |  |
| 10 |  | 15 |  | -2.600 |  | -9.627 |  | 4.428 |  | 2.728 |  | -0.953 |  | 1.000 |  |
|  | | | | | | | | | | | | | | | |
| *Note.*  P-value and confidence intervals adjusted for comparing a family of 7 estimates (confidence intervals corrected using the bonferroni method). | | | | | | | | | | | | | | | |
| *Note.*  Results are averaged over the levels of: speed, bw | | | | | | | | | | | | | | | |

| **Post Hoc Comparisons - speed** | | | | | | | | | | | | | | | |
| --- | --- | --- | --- | --- | --- | --- | --- | --- | --- | --- | --- | --- | --- | --- | --- |
|  | | | | | | **95% CI for Mean Difference** | | | |  | | | | | |
|  | |  | | **Mean Difference** | | **Lower** | | **Upper** | | **SE** | | **t** | | **p _bonf_** | |
| 12 |  | 15 |  | 20.628 |  | 12.277 |  | 28.980 |  | 3.052 |  | 6.758 |  | < .001 |  |
|  |  | 18 |  | 40.220 |  | 31.868 |  | 48.572 |  | 3.052 |  | 13.177 |  | < .001 |  |
|  |  | 21 |  | 57.666 |  | 49.314 |  | 66.018 |  | 3.052 |  | 18.893 |  | < .001 |  |
|  |  | 24 |  | 70.827 |  | 62.458 |  | 79.197 |  | 3.059 |  | 23.156 |  | < .001 |  |
| 15 |  | 18 |  | 19.592 |  | 11.275 |  | 27.908 |  | 3.039 |  | 6.446 |  | < .001 |  |
|  |  | 21 |  | 37.038 |  | 28.721 |  | 45.354 |  | 3.039 |  | 12.186 |  | < .001 |  |
|  |  | 24 |  | 50.199 |  | 41.865 |  | 58.533 |  | 3.046 |  | 16.481 |  | < .001 |  |
| 18 |  | 21 |  | 17.446 |  | 9.129 |  | 25.762 |  | 3.039 |  | 5.740 |  | < .001 |  |
|  |  | 24 |  | 30.607 |  | 22.273 |  | 38.941 |  | 3.046 |  | 10.049 |  | < .001 |  |
| 21 |  | 24 |  | 13.161 |  | 4.827 |  | 21.496 |  | 3.046 |  | 4.321 |  | < .001 |  |
|  | | | | | | | | | | | | | | | |
| *Note.*  P-value and confidence intervals adjusted for comparing a family of 5 estimates (confidence intervals corrected using the bonferroni method). | | | | | | | | | | | | | | | |
| *Note.*  Results are averaged over the levels of: gradient, bw | | | | | | | | | | | | | | | |

| **Post Hoc Comparisons - BW** | | | | | | | | | | | | | | | |
| --- | --- | --- | --- | --- | --- | --- | --- | --- | --- | --- | --- | --- | --- | --- | --- |
|  | | | | | | **95% CI for Mean Difference** | | | |  | | | | | |
|  | |  | | **Mean Difference** | | **Lower** | | **Upper** | | **SE** | | **t** | | **p _bonf_** | |
| 60 |  | 80 |  | -12.503 |  | -18.049 |  | -6.958 |  | 2.360 |  | -5.297 |  | < .001 |  |
|  |  | 100 |  | -19.457 |  | -24.996 |  | -13.918 |  | 2.357 |  | -8.254 |  | < .001 |  |
| 80 |  | 100 |  | -6.953 |  | -12.506 |  | -1.400 |  | 2.363 |  | -2.942 |  | 0.010 |  |
|  | | | | | | | | | | | | | | | |
| *Note.*  P-value and confidence intervals adjusted for comparing a family of 3 estimates (confidence intervals corrected using the bonferroni method). | | | | | | | | | | | | | | | |
| *Note.*  Results are averaged over the levels of: speed, gradient | | | | | | | | | | | | | | | |
