# Supplementary figures and images for: Effect of speed and gradient on plantar force when running on an AlterG® treadmill
Source: BMC Sports Sci Med Rehabil. 2021 Mar 30;13:34. doi: 10.1186/s13102-021-00258-4 (PMC8011121; doi:10.1186/s13102-021-00258-4)

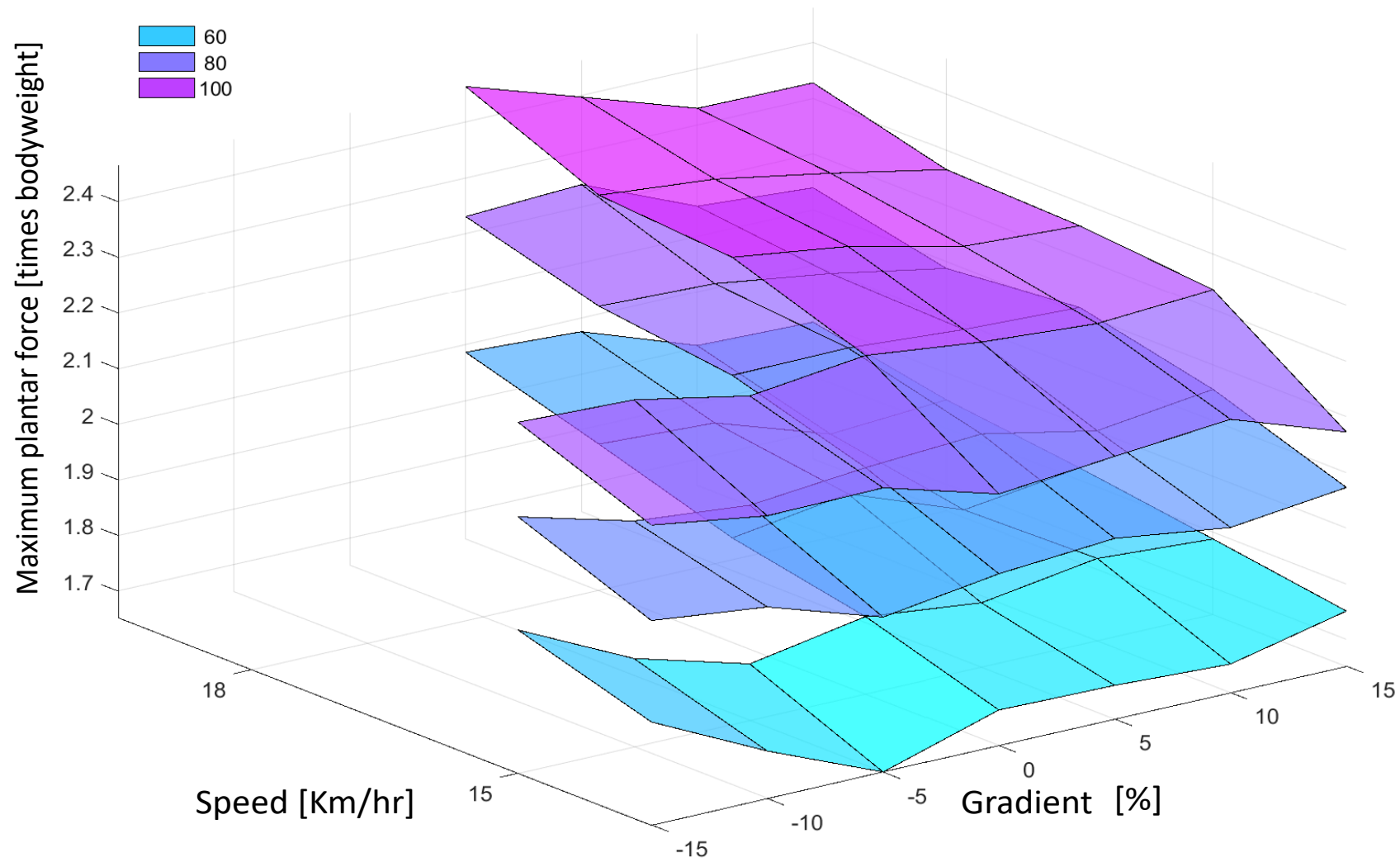

Supplement: Supplementary file 2 — Additional file 2: Figure S1. Multiple linear regression including gradient for Maximum plantar force [times BW] at different running speed [km/hr], gradients [%], and levels of AlterG® assisted bodyweight support [%]. (adj. R2: 0.613, p < 0.001). [file 13102_2021_258_MOESM2_ESM.pdf]

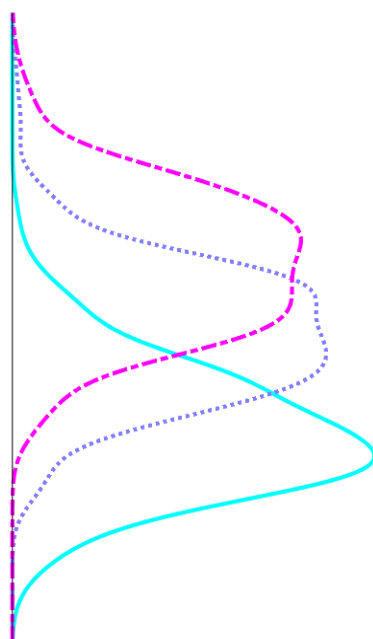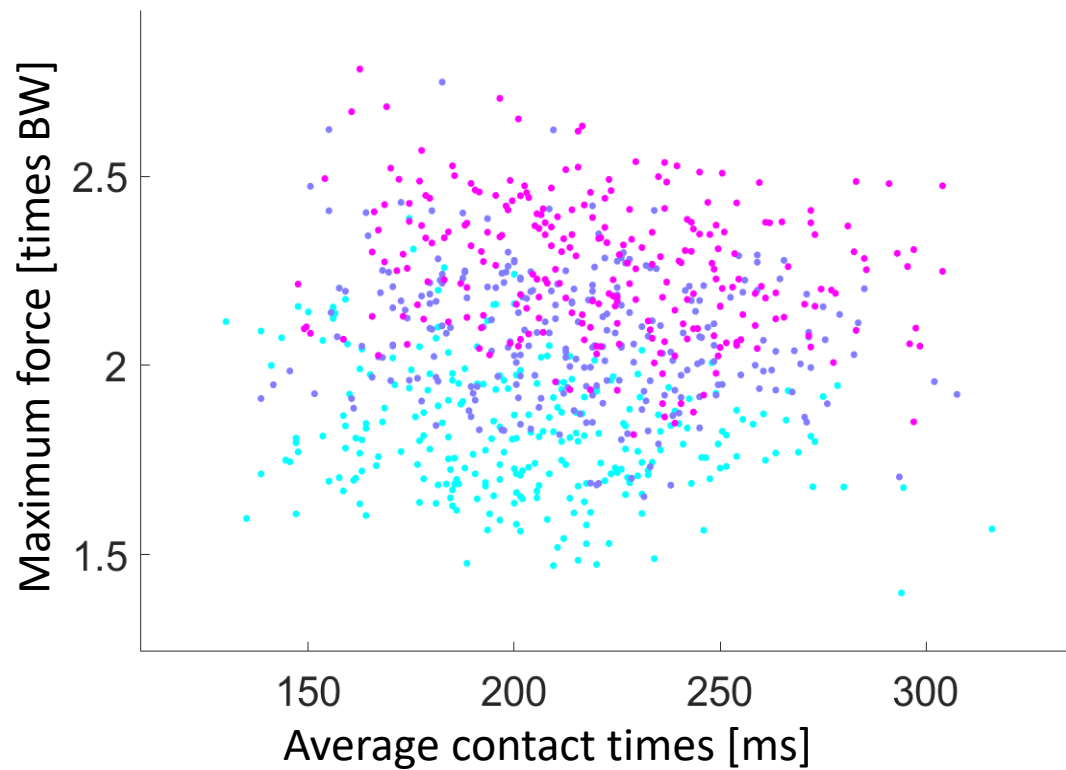

AlterG [%]

- 60
- 80
- 100

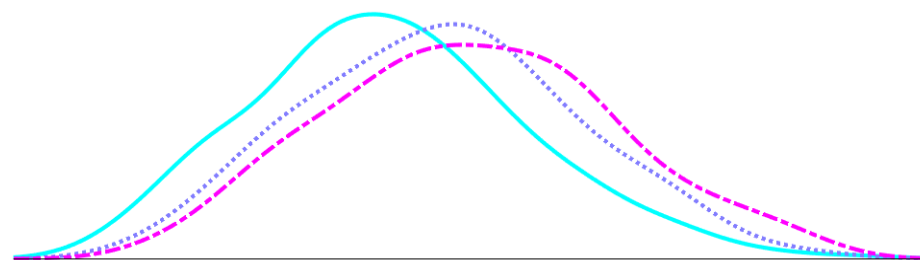

Supplement: Supplementary file 3 — Additional file 3: Figure S2. Individual data for maximum plantar force [times BW] and contact times at different AlterG® assisted bodyweight support. [file 13102_2021_258_MOESM3_ESM.pdf]

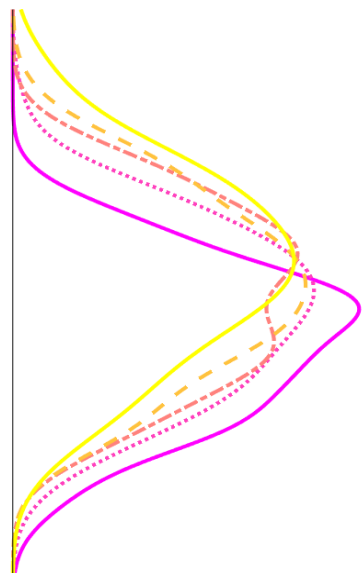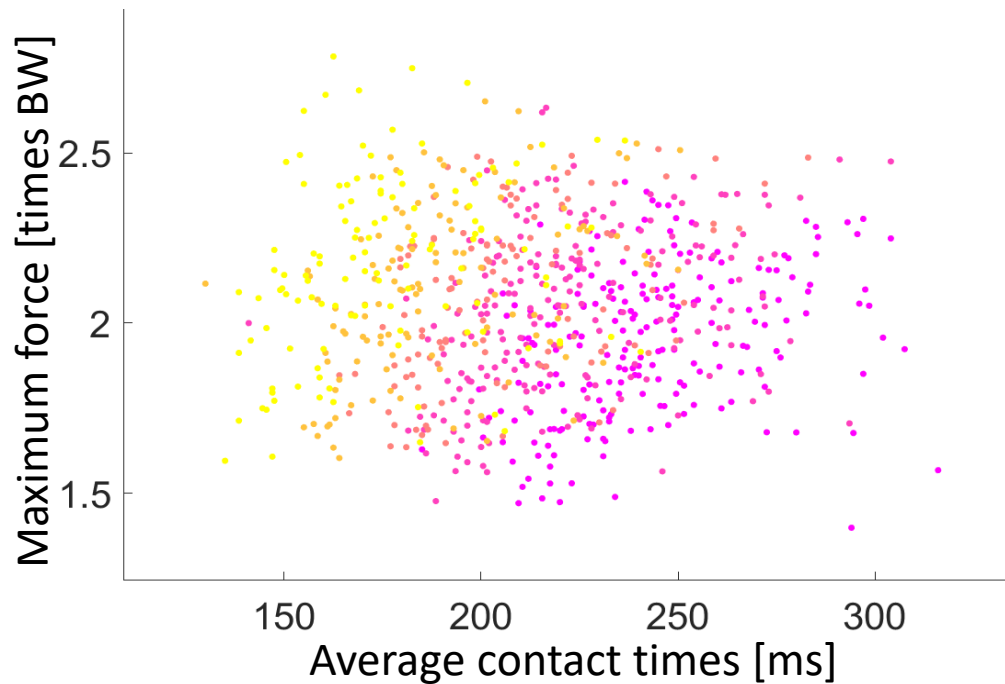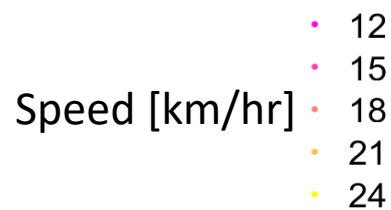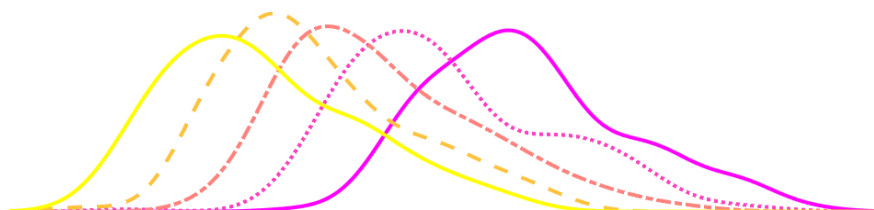

Supplement: Supplementary file 4 — Additional file 4: Figure S3. Individual data for maximum plantar force [times BW] and contact times at different running speeds. [file 13102_2021_258_MOESM4_ESM.pdf]

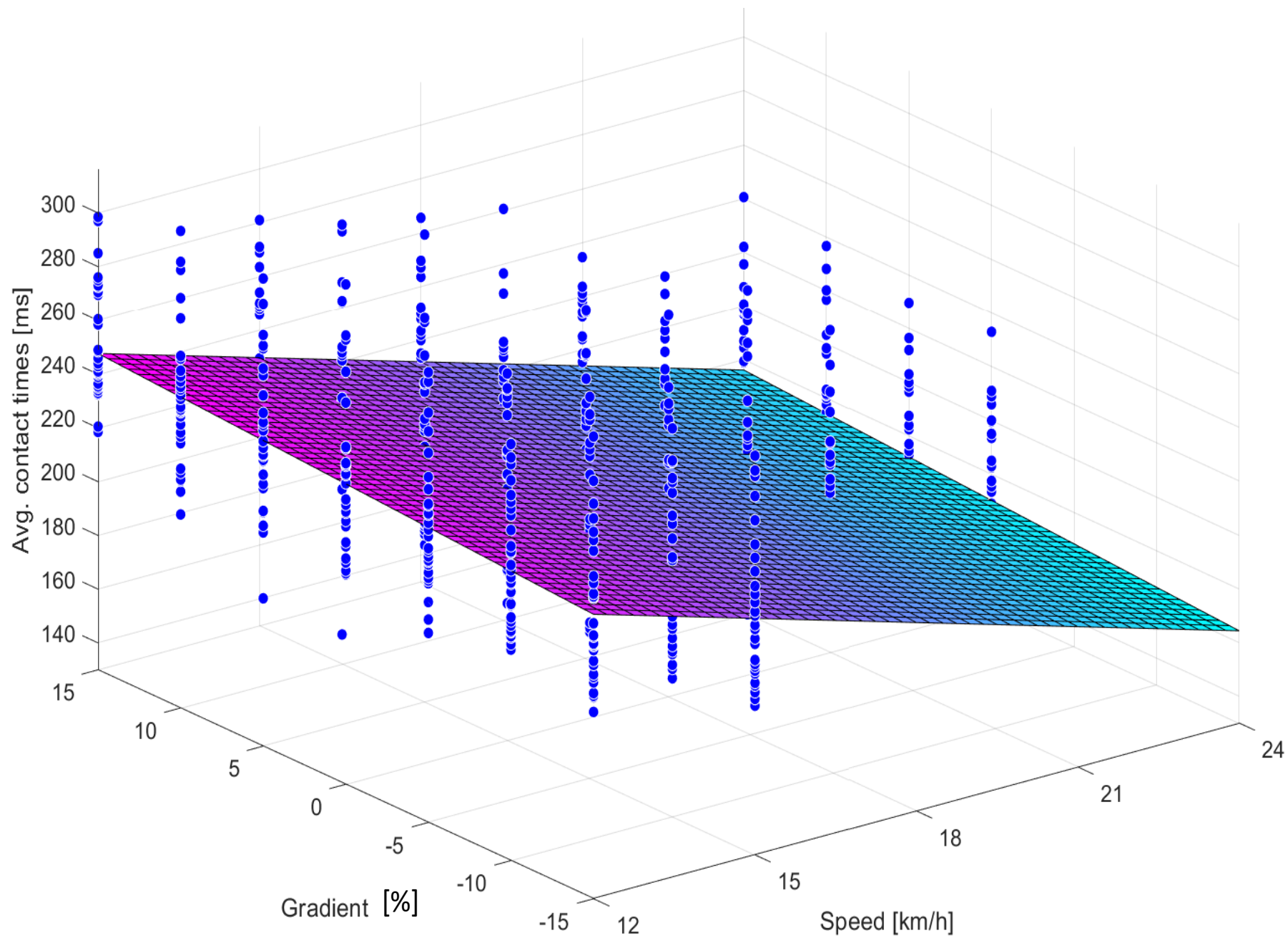

Supplement: Supplementary file 5 — Additional file 5: Figure S4. Multiple linear regression including gradient for average contact times. (adj. R2: 0.52, p < 0.001). [file 13102_2021_258_MOESM5_ESM.pdf]
